# Sociodemographic and mental health characteristics associated with changes in movement behaviours due to the COVID-19 pandemic in adolescents

**DOI:** 10.1186/s44167-022-00004-2

**Published:** 2022-10-02

**Authors:** Amanda Lien, Hugues Sampasa-Kanyinga, Karen A. Patte, Scott T. Leatherdale, Jean-Philippe Chaput

**Affiliations:** 1 Temerty Faculty of Medicine, University of Toronto, ON Toronto, Canada; 2 School of Epidemiology and Public Health, University of Ottawa, Ottawa, ON Canada; 3 Department of Health Sciences, Brock University, Niagara Region, St. Catharines, ON Canada; 4 School of Public Health Sciences, University of Waterloo, Waterloo, ON Canada; 5 Healthy Active Living and Obesity Research Group, Children’s Hospital of Eastern Ontario Research Institute, Ottawa, ON Canada

**Keywords:** Physical activity, Sleep, Screen time, COVID-19, Mental health, Adolescent health

## Abstract

**Objectives:**

Control measures enacted to control the spread of COVID-19 appear to have impacted adolescent movement behaviours. It remains unclear how these changes relate to sociodemographic characteristics and indicators of mental health. Understanding these relationships can contribute to informing health promotion efforts. The purpose of this study is to examine sociodemographic and mental health characteristics associated with changes in movement behaviours (physical activity, screen time, sleep duration) due to the COVID-19 pandemic among adolescents.

**Methods:**

This cross-sectional study used May–June 2020 survey data and included 7349 students from Quebec, Ontario, and British Columbia (Canada). ANOVA, χ^2^ tests, and estimation of effect sizes using Cohen’s d and h tests were performed between self-reported perceived changes (increase; decrease; no change) to physical activity, TV watching, social media use, and sleep duration as a result of the COVID-19 pandemic and gender, age, race/ethnicity, income, depression and anxiety symptoms, flourishing-languishing, and self-rated mental health.

**Results:**

Over half of students reported increases in TV viewing and social media use and approximately 40% reported decrease in physical activity and increase in sleep duration due to the COVID-19 pandemic. More females (68.9%) than males (54.3%) reported increase in social media use (Cohen’s h ≥ 0.2–0.5). No change from pre-COVID-19 social media use and sleep duration were associated with fewer depression and anxiety symptoms and better self-rated mental health compared to reports of an increase or decrease. These effect sizes ranged from small-to-moderate to moderate-to-large (Cohen’s d/h ≥ 0.2–0.8). Decreased physical activity and sleep duration were associated with better psychological functioning with effects sizes of small-to-moderate. Compared to an increase or no change, decreased sleep had the largest effect size of less frequent depression symptoms (Cohen’s d ≥ 0.5–0.8).

**Conclusion:**

Maintaining pre-COVID-19 screen time and sleep duration during early stages of the COVID-19 lockdown was generally beneficial to mental health, with sleep being particularly important in regards to symptoms of depression. Psychological functioning was more related to physical activity and sleep than screen time during the pandemic.

## Background

Substantial control measures have been implemented across the world to reduce the spread of coronavirus disease (COVID-19), a novel disease caused by severe acute respiratory syndrome coronavirus 2 (SARS-CoV-2). The potential implications of these preventative measures on mental and physical health are far-reaching and pose an unprecedented challenge to public health. In particular, there are growing concerns regarding the potential effects of restrictions on children and adolescents [1, 2]. In Canada, notable restrictions include closure of schools in the spring of 2020 followed by States of Emergency being declared days after, which further restricted various activities including organized sport and usage of recreational facilities. Such restrictions did not significantly relax until spring of the following year [3].

Current evidence based on cross-sectional studies indicate that prevalence of symptoms of anxiety and depression during the early phases of the pandemic was high, particularly among adolescents [4]. In Canada specifically, prospective studies found that the initial phase of COVID-19 lockdown measures appeared to be detrimental to mental health among adults [5, 6] but not to the general adolescent population [7]. It has been well-established that meeting the Canadian 24-h movement guidelines for adolescents, which include engaging in at least 60 min of physical activity (PA), obtaining 8–10 h of sleep, and limiting screen time to 2 h or less per day, are associated with various health benefits, including those of mental health [8, 9]. A scoping review of searches conducted in November 2020 and January 2021 highlights the impact that the COVID-19 pandemic and related restrictions has had on movement behaviours in children and youth in 40 countries [10]. Overall, it consistently found reports of decreases in PA duration, with nearly all studies finding a decrease in the proportion of youth and children meeting the PA guideline. That being said, there were also some groups that reported stability or increase in unstructured PA. In terms of screen time, notable increases were observed compared to pre-pandemic durations, with increases being observed in both academic-related and leisure-time usage. All studies that considered screen time guidelines found an increase in the proportion of children exceeding the recommended amount of screen time. In contrast to the fairly consistent PA and screen time findings, the impact of the COVID-19 pandemic on sleep duration was more variable, where increases, decreases, and stability were observed in different studies. There was also inconsistency in the change in proportion of children meeting the sleep guideline. However, decrease in sleep quality and increase in sleep problems were commonly reported.

Our understanding of the COVID-19 pandemic’s impact on movement behaviours and mental health is constantly developing and a consensus of its impact remains elusive. To our knowledge, it also remains largely unknown which sociodemographic and mental health characteristics are linked with perceived changes (increase; decrease; no change) to the different movement behaviours during the pandemic. Identifying characteristics associated with pandemic-related changes to movement behaviours allows for an opportunity for outreach to populations of adolescents that are particularly susceptible to poor adherence to the 24-h movement guidelines and potential related mental health impacts. Determining which movement behaviours changes may be protective or detrimental to mental health can better inform and support health promotion and primary prevention efforts as we progress through and eventually out of the pandemic.

Thus, the purpose of this study was to describe the perceived changes in PA, recreational screen time, and sleep duration due to the COVID-19 pandemic among adolescents and examine the relationship between these changes and various sociodemographic and mental health characteristics. We hypothesized that decreased PA, increased screen time, and increased sleep duration would be reported and that better mental health characteristics would be observed with increased PA, decreased screen time, and increased sleep duration.

## Methods

### Design

The current observational, cross-sectional study used student- and school-level data from May and June 2020 of the Cannabis use, Obesity, Mental health, Physical activity, Alcohol use, Smoking, and Sedentary behaviour (COMPASS) study. COMPASS is an ongoing prospective cohort study that began in 2012–2013 and collects survey data on the health behaviours of Canadian secondary school students on an annual basis. The student questionnaire is administered annually and is available to be completed during a 2-week timeframe for each cycle. Schools permitting active-information, passive-consent parental permission protocols were selected to participate in the COMPASS study, which are critical for collecting robust youth mental health data [11].

In 2020, as schools were closed for in-person learning due to the COVID-19 pandemic response, surveys were conducted online using Qualtrics XM online survey software (Qualtrics, Provo, UT, USA). A survey link was emailed to all students by their schools, followed by a reminder email 1 week after [12]. The majority of initial emails were sent out on May 12, 2020; the earliest school email was sent May 1, 2020. Ontario and British Columbia surveys were left open for 2 weeks after the initial email, and Quebec surveys for 4 weeks; the last survey closed on July 6, 2020. All students attending participating schools were eligible to participate and could withdraw at anytime. During the early COVID-19 lockdown (spring 2020), 9630 secondary school students from 51 schools across Quebec, Ontario, and British Columbia completed the survey. Missing data was handled with listwise deletion of incomplete cases, resulting in reduction of the sample size to 7349 students.

All procedures received ethics approval from the University of Waterloo (ORE#30118), Brock University (REB#18-099), CIUSSS de la Capitale-Nationale–Université Laval (#MP-13-2017-1264), and participating school boards. Further details regarding the COMPASS study design and implementation can be accessed in print [13] and online (www.compass.uwaterloo.ca).

### Measures

#### Student-level sociodemographic characteristics and mental health

Student-level variables included gender (male, female, other), race/ethnicity (White, Asian, Black, Latin American/Hispanic, Other/Mixed), age (11–19 years old), and measures of mental health, which included self-reported symptoms of depression and anxiety, overall psychological functioning, and overall mental health. Socioeconomic status (SES) was a school-level variable derived from the median household income of census divisions corresponding to school postal codes described by the 2016 Census [14]. To attain sufficient power, race/ethnicity was dichotomized into “White” and “Non-White”, the SES categories were collapsed into three categories, “high”, “medium”, and “low” based on the Canadian median income, and the “other” category for gender was omitted.

To assess symptoms of depression, the COMPASS questionnaire employed the Center for Epidemiologic Studies Depression Scale (Revised)-10 (CESD-R-10). The CESD-R-10 is a 10-item scale that describes the frequency of experiencing symptoms of depression, such as feeling sad, hopeless, unmotivated, and apathetic [15]. Construct and structural validity have been demonstrated with use of this scale in the adolescent population [16]. Scores can range from 0 to 30, with higher scores representing greater frequency of depression symptoms. Symptoms of anxiety were assessed with the Generalised Anxiety Disorder 7-item Scale (GAD-7), which describes the frequency of experiencing symptoms such as trouble relaxing, restlessness, and uncontrollable worrying [17]. The scale has demonstrated construct validity among adolescents [18]. Scores can range from 0 to 21, with higher scores representing greater frequency of anxiety symptoms. In addition, the survey used Diener’s Flourishing Scale (FLOURISH) to measure overall psychological functioning, which assesses perceptions of success in various aspects of life, such as relationships, self-esteem, interest in daily activities, and optimism [19]. Validity of the 8-item FLOURISH scale has been demonstrated in adolescents [20, 21]. The 7-point Likert scale was modified to a 5-point scale to be more suitable for large, school-based studies such that scores can range from 8 to 41, with higher scores representing poorer psychological functioning [22]. Previously established internal consistency of the CESD-R-10, GAD-7, and FLOURISH scales were high (α_CESD-R-10_ = 0.82, α_GAD-7_ = 0.90, α_FLOURISH_ = 0.94). Lastly, self-rated mental health was assessed by the single-item question, “In general, how would you rate your mental health?” Response options ranged from “excellent” to “poor” on a 5-point scale, with higher scores representing poorer mental health. The concept of self-rated health, commonly used in population-based surveys, is considered an appropriate measure of adolescent health by the World Health Organization and has been shown to have good psychometric properties among adolescents [23].

#### Movement behaviours

Change in movement behaviours as a result of the COVID-19 pandemic were assessed with the following questions: (1) “How has your life changed because of COVID-19 in terms of physical activity?”, (2) “How has your life changed because of COVID-19 in terms of time spent watching TV/movies or playing video games?”, (3) “How has your life changed because of COVID-19 in terms of time spent surfing/posting on social media?”, (4) “How has your life changed because of COVID-19 in terms of sleep?”. For each of the questions, possible self-reported answer options included “increased”, “stayed the same/not applicable”, or “decreased”. The question regarding PA captures a general and subjective sense of PA given that intensity or type of activity was not specified in the question. Similarly, the term “social media” was not further elaborated on and would therefore encompass any internet-based form of communication that participants engaged with.

### Statistical analysis

All analyses were conducted using R 4.0.2 [24]. Descriptive analyses were conducted with analysis of variance (ANOVA) and χ^2^ tests to examine the relationship between each sociodemographic characteristic or mental health measure and change in each movement behaviour due to the COVID-19 pandemic (increase; decrease; no change). Statistical significance was defined as *p* < 0.05. For significant ANOVA and χ^2^ tests with more than two categories, post hoc pairwise comparisons were performed to determine which category or categories demonstrated statistically significant differences. Post hoc Cohen’s d and h tests were also performed on significant relationships to quantify effect size between means and proportions, respectively. Very small, small to moderate, moderate to large, and very large effect sizes were defined as ≤ 0.2, > 0.2–0.5, > 0.5–0.8, > 0.8, respectively. Very small effect sizes were considered negligible. Comparison of the sociodemographic characteristics and mental health measures of incomplete versus complete cases was also performed via Student’s t tests and tests of proportions followed by estimation of effect sizes via Cohen’s d and h tests.

## Results

Students excluded due to missing data were significantly more likely to report poorer psychological functioning and to be Non-White, compared to those included in the analytical sample. The effect size in the difference of psychological functioning between students with complete data and excluded students due to missing data was very small and negligible (Cohen’s d ≤ 0.2) and that of ethnicity was small (Cohen’s d ≥ 0.2–0.5).

Characteristics of the sample are summarized in Table 1. Over half of the sample identified as female and over three quarters reported being of White ethnoracial background. Most students were from Quebec (63.2%) and Ontario (32.0%). In terms of movement behaviours, a majority of students reported an increase in TV watching (76.2%) and social media use (63.2%) as a result of the COVID-19 pandemic. Meanwhile, 39.1% of students reported a decrease in PA whereas 30.3% reported an increase in PA. In terms of sleep duration, 23.5% reported a decrease and 41.7% reported an increase in sleep duration as a result of the pandemic.

**Table 1:** Sample’s sociodemographic and mental health characteristics and movement behaviour changes during COVID-19 pandemic (May–June 2020)

N = 7349	% or mean (SD)
Age (years)	15.0 (1.5)
Gender	
Female	61.3
Male	36.9
Other	0.9
Mental health	
Excellent	24.7
Very good	31.6
Good	24.4
Fair	13.8
Poor	5.5
Race/ethnicity	
White	78.0
Asian	6.8
Black	3.2
Latin American/Hispanic	1.6
Other/mixed	10.3
Median income ($)	
25,000–50,000	15.0
50,001–75,000	61.1
75,001–100,000	22.5
> 100,000	1.4
CESD-R-10^a^	8.9 (6.2)
GAD-7^b^	6.2 (5.5)
FLOURISH^c^	32.7 (5.6)
TV viewing	
Increase	76.2
Decrease	3.7
No change	20.1
Social media	
Increase	63.2
Decrease	5.2
No change	31.6
PA	
Increase	30.3
Decrease	39.1
No change	30.6
Sleep	
Increase	41.7
Decrease	23.5
No change	34.8

*CESD-R-10* Center for Epidemiologic Studies Depression Scale (Revised)-10, *GAD7* Generalised Anxiety Disorder 7-item Scale, *FLOURISH* Diener’s Flourishing Scale

^a^Scale ranging from 0 to 30 measuring depression symptoms where higher scores represent greater frequency of symptoms

^b^Scale ranging from 0 to 21 measuring anxiety symptoms where higher scores represent greater frequency of symptoms

^c^Scale ranging from 8 to 41 measuring psychological functioning where higher scores represent poorer functioning

χ^2^ and ANOVA test findings are shown in Tables 2 and 3, respectively. On average, students who reported an increase in social media use were older than those who reported a decrease or no change as a result of the COVID-19 pandemic. Students who reported a decrease in PA were also older than those who reported an increase or no change. Students that reported an increase or decrease in sleep duration were older than those that reported no change. Male students were more likely to report an increase (79.1%) in TV watching compared to their female counterparts (74.7%) while female students (68.9%) were more likely to report an increase in social media use compared to male students (54.3%). Female students were also more likely to report an increase in PA (32.0%) compared to male students (27.7%) and a decrease in sleep (25.1%) compared to male students (20.3%). Non-White students were more likely to report a decrease in PA (43.7%) compared to White students (37.8%) and an increase in sleep (45.7%) compared to White students (40.5%). White students were also more likely to report a decrease in social media (7.0%) compared to Non-White students (4.7%). Lastly, students from medium (63.8%) and low (65.9%) median income areas were more likely to report an increase in social media use compared to those in high median income areas (60.1%) while those from low median income areas (23.3%) were more likely to report no change in TV watching compared to those in high (20.1%) and medium (19.3%) income areas. Although significant, effect sizes in all relationships were very small and negligible (Cohen’s h ≤ 0.2), with the exception of increased social media use being reported more frequently among females compared to males (Cohen’s h ≥ 0.2–0.5).

**Table 2:** χ^2^ tests of movement behaviour changes during COVID-19 pandemic and sociodemographic characteristics (May–June 2020)

			Proportion (%)			χ^2^ value	*p* value
			Increase	Decrease	No change		
Gender	TV	Female	74.7	3.8	21.5	18.64	< 0.001
		Male	79.1	3.4	17.5		
	Effect size^a^		VS	–	VS		
	Social media	Female	68.9	4.5	26.6	156.57	< 0.001
		Male	54.3	6.2	39.5		
	Effect size^a^		SM	VS	SM		
	Physical activity	Female	32.0	39.1	28.9	20.70	< 0.001
		Male	27.7	39.0	33.3		
	Effect size^a^		VS	–	VS		
	Sleep	Female	43.0	25.1	31.9	49.10	< 0.001
		Male	40.1	20.3	39.6		
	Effect size^a^		–	VS	VS		
Ethnicity	TV	White	75.7	3.6	20.7	7.79	0.02
		Non-White	77.8	4.3	17.8		
	Effect size^a^		–	–	–		
	Social media	White	63.1	4.7	32.2	16.16	< 0.001
		Non-White	63.6	7.0	29.4		
	Effect size^a^		–	VS	–		
	Physical activity	White	30.8	37.8	31.4	18.62	< 0.001
		Non-White	28.5	43.7	27.8		
	Effect size^a^		–	VS	VS		
	Sleep	White	40.5	23.0	36.5	31.30	< 0.001
		Non-White	45.7	25.3	29.0		
	Effect size^a^		VS	–	–		
Income	TV	High	75.5	4.4	20.1	12.32	0.02
		Medium	77.2	3.5	19.3		
		Low	73.2	3.5	23.3*		
	Effect size^a^		–	–	VS		
	Social media	High	60.1*	6.0	33.9	12.80	0.01
		Medium	63.8	5.1	31.1		
		Low	65.9	4.4	29.7		
	Effect size^a^		VS	–	–		
	Physical activity	High	32.4	38.8	28.8	9.63	0.05
		Medium	30.0	38.6	31.3		
		Low	27.8	41.5	30.7		
	Effect size^a^		–	–	–		
	Sleep	High	43.9	23.2	32.9	5.53	0.24
		Medium	41.0	23.5	35.5		
		Low	40.8	23.8	35.4		
	Effect size^a^		–	–	–		

*Value that significantly varies from others in three-way comparisons

^a^Pairwise *p* value between sociodemographic categories < 0.05, where VS, SM, ML, VL represent very small (≤ 0.2), small-to-moderate (> 0.2–0.5), moderate-to-large (> 0.5–0.8), and very large (> 0.8) Cohen’s h or d effect sizes respectively

**Table 3:** ANOVA of movement behaviour changes during COVID-19 pandemic, age, and mental health indicators (May–June 2020)

		Mean, SD			Effect size^a^	F value	*p* value
		Increase	Decrease	No change			
Age	TV	15.07, 1.54	14.88, 1.62	14.98, 1.55	–	3.83	0.02
	Social media	15.17, 1.52	14.86, 1.56	14.82, 1.58	I-D: VS; I-NC: VS	42.23	< 0.001
	PA	14.98, 1.51	15.15, 1.54	14.98, 1.59	I-D: VS; D-NC: VS	11.34	< 0.001
	Sleep	15.14, 1.56	15.07, 1.53	14.92, 1.54	I-NC: VS; D-NC: VS	14.39	< 0.001
CESD-R-10^a^	TV	9.01, 6.15	9.79, 7.24	8.45, 6.09	I-NC: VS; D-NC: VS	7.49	< 0.001
	Social media	9.58, 6.33	9.63, 6.74	7.51, 5.55	I-NC: SM; D-NC: SM	91.10	< 0.001
	PA	8.46, 5.95	9.78, 6.43	8.29, 5.98	I-D: VS; D-NC: VS	46.26	< 0.001
	Sleep	8.06, 5.61	12.15, 6.82	7.79, 5.64	I-D: ML; D-NC: ML	335.70	< 0.001
GAD-7^b^	TV	6.20, 5.49	7.23, 6.34	5.90, 5.58	I-D: VS; D-NC: VS	6.87	0.001
	Social media	6.68, 5.63	6.90, 6.27	5.06, 5.07	I-NC: SM; D-NC: SM	70.74	< 0.001
	PA	6.09, 5.45	6.55, 5.61	5.80, 5.52	I-D: VS; D-NC: VS	11.80	< 0.001
	Sleep	5.59, 5.17	8.48, 6.23	5.32, 5.04	I-D: VS; D-NC: SM	20.40	< 0.001
FLOURISH^c^	TV	32.57, 5.54	32.93, 6.51	33.15, 5.67	I-NC: VS	6.41	0.002
	Social media	32.54, 5.55	32.07, 6.53	33.14, 5.53	I-NC: VS; D-NC: VS	11.53	< 0.001
	PA	33.57, 5.35	31.88, 5.73	32.91, 5.56	I-D: SM; D-NC: VS; I-NC: VS	60.10	< 0.001
	Sleep	33.31, 5.27	30.97, 6.03	33.15, 5.48	I-D: SM; D-NC: SM	112.20	< 0.001
Global mental health	TV	2.45, 1.15	2.59, 1.31	2.36, 1.17	I-NC: VS; D-NC: VS	5.27	0.005
	Social media	2.53, 1.16	2.53, 1.31	2.23, 1.12	I-NC: SM; D-NC: SM	55.93	< 0.001
	PA	2.36, 1.16	2.57, 1.18	2.35, 1.12	I-D: VS; D-NC: VS	28.65	< 0.001
	Sleep	2.34, 1.12	2.87, 1.23	2.27, 1.10	I-D: SM; D-NC: SM; I-NC: VS	148.57	< 0.001

*CESD-R-10* Center for Epidemiologic Studies Depression Scale (Revised)-10, *GAD7* Generalised Anxiety Disorder 7-item Scale, *FLOURISH* Diener’s Flourishing Scale

^a^Scale ranging from 0 to 30 measuring depression symptoms where higher scores represent greater frequency of symptoms

^b^Scale ranging from 0 to 21 measuring anxiety symptoms where higher scores represent greater frequency of symptoms

^c^Scale ranging from 8 to 41 measuring psychological functioning where higher scores represent poorer functioning

^d^Pairwise *p* value < 0.05, where VS, SM, ML, VL represent very small (≤ 0.2), small-to-moderate (> 0.2–0.5), moderate-to-large (> 0.5–0.8), and very large (> 0.8) Cohen’s h or d effect sizes and I-D, D-NC, I-NC represent increase to decrease, decrease to no change, and increase to no change pairwise comparisons, respectively

Students that reported no change to their TV watching reported better self-rated mental health and less frequent symptoms of depression (Cohen’s d/h ≤ 0.2) compared to those that increased or decreased their TV watching (Cohen’s d/h ≤ 0.2). A similar relationship was seen with social media use, where students that reported no change to their social media use reported better self-rated mental health (Cohen’s h ≥ 0.2–0.5) and less frequent symptoms of depression (Cohen’s d ≥ 0.2–0.5) compared to those that increased or decreased their social media use. However, the relationship between these outcomes with social media use had larger effect sizes than with TV watching. Similarly, students that reported no change in their social media use reported less frequent symptoms of anxiety than their peers that reported an increase or decrease (Cohen’s d ≥ 0.2–0.5). Interestingly, students that decreased their TV watching reported more symptoms of anxiety compared to those that increased or did not change their TV watching, although the effect size was very small and negligible (Cohen’s d ≤ 0.2). Students that reported an increase or no change in PA had better self-rated mental health and fewer symptoms of depression and anxiety compared to those that reported decreased PA, although the effect size was very small and negligible (Cohen’s d ≤ 0.2). Similarly, students that reported increased or no change in their sleep had better self-rated mental health (Cohen’s h ≥ 0.2–0.5) and fewer symptoms of depression (Cohen’s d ≥ 0.5–0.8) and anxiety (Cohen’s d_increase_ ≤ 0.2; Cohen’s d_no change_ ≥ 0.2–0.5) compared to those that decreased their sleep. Decreased sleep had a moderate-to-large effect size in terms of increased frequency of depression symptoms (Cohen’s d ≥ 0.5–0.8).

Better psychological functioning scores were observed with reports of increased TV watching compared to decreased TV watching. Better scores were also observed with increased or decreased social media use compared to no change. The effect sizes of these findings were very small (Cohen’s d ≤ 0.2). Compared to those that reported decreases or no change in PA, those that increased their PA had poorer psychological functioning scores (Cohen’s d_decrease_ ≥ 0.2–0.5; Cohen’s d_no change_ ≤ 0.2). Lastly, compared to students that reported decreased sleep, those that reported increased or no change in sleep reported negligibly poorer psychological functioning (Cohen’s d ≤ 0.2).

## Discussion

Ongoing efforts continue to be made to understand the potential impacts of COVID-19-related preventative public health measures. This large cross-sectional study is among the first to describe the sociodemographic and mental health characteristics of Canadian adolescents in relation to perceived changes in movement behaviours during the months immediately following introduction of lockdown measures. The survey capturing responses of students across Ontario, Quebec, and British Columbia (Canada) found that a perceived increase in screen time as a result of the pandemic was highly prevalent, with social media increase among females compared to males having the largest effect size among gender-based comparisons of movement behaviour changes. This finding aligns with studies that have observed greater use of social media among females compared to males during the COVID-19 pandemic for reasons such as to cope with social isolation [25, 26]. Many adolescents also perceived a decrease in their PA and an increase in sleep due to the COVID-19 pandemic. Perceived changes to social media usage and sleep due to the COVID-19 pandemic were related to poorer self-rated mental health and symptoms of depression and anxiety while a perceived increase in PA and sleep were related to poorer perceptions of psychological functioning. Although significant differences in perceived movement behaviour changes related to age, race/ethnicity, gender, and income were observed, they were very small and therefore unlikely to be clinically relevant. Replication analysis with additional data and studies using a longitudinal design are needed to account for the fluctuating course of the pandemic and related preventative measures.

In line with our findings, a narrative literature review of studies conducted in various countries has identified a rising trend of screen time during the COVID-19 pandemic [27]. Similarly, our finding of increased sleep duration among over 40% of adolescents during the COVID-19 pandemic also agrees with the current literature [10], although over one-fifth perceived a decrease in their sleep. That being said, our study did not explore changes to sleep quality, which evidence suggests has become poorer during the pandemic [10, 28]. Our results for PA align with previous studies indicating that its duration has decreased among many youth, although 30% of students in our study perceived an increase during the pandemic. The impact may vary by region given differences in levels and timing of restrictions and the unique contextual factors of individual countries and communities [10, 29]. In Canada, evidence suggests that PA has decreased among children and youth [30].

In general, impacts that COVID-19-related preventative health measures may be having on movement behaviours is of significant concern considering their relationship with physical and mental health. To demonstrate, obtaining adequate amounts of PA and sleep and limiting screen time are strongly linked with a wide range of benefits, including better physical, social, and emotional health indicators among all age groups, including children and adolescents [8]. Healthy movement behaviours are also related to important predictors of positive mental health, with sleep playing a particularly important role [9]. In the context of the COVID-19 pandemic, our study also finds that decreased sleep is, compared to other movement behaviours, most strongly linked with more frequent symptoms of depression. This finding aligns with the previously established consistent relationship between short sleep duration and depression [31]. However, we also found that those reporting no changes to social media use or sleep demonstrated better overall mental health and fewer depression and anxiety symptoms compared to those that reported an increase or decrease. This phenomenon might be explained by considering the reciprocal nature of the relationship between mental health and movement behaviours. Firstly, individuals that reported an increase in social media use or reduction in sleep may have experienced more frequent symptoms, consistent with previous literature [8, 9]. This also appears to agrees with a Belgian study that observed that increasing social media use can be a coping mechanism for adolescents that endorse feeling anxious or lonely during the COVID-19 pandemic [32]. Individuals reporting no change and those reporting fewer symptoms may have also been less vulnerable to impacts of the pandemic and were therefore able to maintain their pre-COVID-19 levels of screen time and sleep. In addition, both short and long sleep duration have been shown to be associated with negative mental health compared to normal duration and can be symptoms of depression [33]. Furthermore, individuals that reduced their social media use may have also lost out on the benefits that it can provide during socially isolating times, including opportunities for relational interactions and self-expression [34].

The unique circumstances of the pandemic may also explain the counterintuitive finding that perceptions of increased PA and sleep were related to poorer psychological functioning, which is defined as less perceived success in various aspects of within-self and external life, such as one’s relationships with others and self-esteem. Prior to the COVID-19 pandemic, better psychological functioning was observed with obtaining at least 60 min of PA and 8–10 h of sleep per day [35]. Our findings may reflect the relationship of psychological functioning with obtaining adequate but also excessive amounts of sleep during the pandemic, given that overtraining has been previously observed prior to the pandemic to be related to negative psychological indicators and disorders such as anorexia nervosa [36, 37]. Furthermore, it is possible that students used PA as a coping mechanism for psychological distress. With restrictions put in place to limit group activity, PA may have been more likely to be a solitary endeavor and could have made individuals feel more isolated if performed in excess, which in itself can also generate psychological symptoms [36]. Furthermore, individuals with greater body image-related concerns, compounded by an increase in social media use and less structure during the pandemic, might engage in more PA and be at an increased risk of depression and anxiety symptoms and lower psychological functioning, especially during the early period of heavier restrictions. However, larger scale and longitudinal studies are required to assess this hypothesis.

Overall, the current study’s findings support population-based efforts to encourage adolescents to obtain adequate yet non-excessive amounts of PA and sleep and to limit their screen time within reason to support their well-being while public health restrictions and/or lockdown measures are in place. Although our findings suggest a positive relationship between maintaining pre-COVID-19 movement behaviours and various measures of mental health, several of the effects size were small and further studies are required to establish any cause-and-effect relationships. Given the everchanging tightening and loosening of restrictions, further longitudinal studies should assess the within-individual effects of movement behaviours and sociodemographic and mental health characteristics over longer periods of time during the pandemic.

The major strengths of this study include its large sample size, the inclusion of multiple Canadian provinces, the well-validated mental health measures, and negligible variability between those included and excluded from the study’s analysis due to completeness of data. Limitations to consider include an inability to infer causality due to the cross-sectional design of the study as well as potential bias associated with self-reporting of changes to movement behaviours. In addition, the lack of specification of PA intensity in the survey question likely resulted in different interpretations of what PA refers to and therefore introduces variability that may have influenced results. Future studies would employ a question that specifies intensity such as moderate-to-vigorous, to not only ensure more standardization but also to capture the amount of PA that is more clinically relevant. While there is established acceptable reliability and validity of self-reported movement behaviour durations in adolescents, it should be noted that perceived changes to movement behaviours may be lacking in validity as proxy of true change in movement behaviour durations, such that findings of this study should interpreted with this distinction in mind [38–40]. Data used in the current study also only captured the early stage of the COVID-19 pandemic and cannot therefore be extrapolated to apply to other phases of the pandemic, though future waves of COMPASS data will allow for this exploration both longitudinally and via repeated cross-sectional studies. Given that sociodemographic characteristics and mental health indicators are often at interplay with one another in actuality, the findings of this study should also be interpreted with caution as analyses were conducted separately for each sociodemographic characteristic and mental health indicator. Furthermore, results may not be generalizable to all Canadian adolescents, as data were collected via an online survey and the COMPASS study was not designed to be representative.

## Conclusion

Maintaining pre-COVID-19 social media use and sleep were linked to less frequent depression and anxiety symptoms, while increased sleep and PA were linked to poorer psychological functioning. Although significant differences were found between movement behaviour changes and sociodemographic characteristics, the effect sizes were negligible, with the exception of increased social media use among females compared to males. The largest effect size was observed between changes in sleep and depression symptoms, where maintenance as opposed to an increase or decrease conferred the least frequent symptoms, highlighting the important relationship between appropriate sleep duration and mental well-being. Future work could include replication studies with a longitudinal design and greater segmentation of movement behaviour categories to included excessive sleep, PA, and limits on screen time. The important public health implications of our findings relate to the modifiable nature of movement behaviours and their notable influence on mental health. Therefore, stakeholders such as public health authorities, health service providers, schools, parents, and adolescents should be aware of the role of public health policies and restrictions on movement behaviours in health promotion efforts.

## Data Availability

Researchers can contact the University of Waterloo’s Office of Research Ethics (ohrac@uwaterloo.ca) for access to the data.

## Abbreviations

COVID-19: Coronavirus disease
SARS-CoV-2: Severe acute respiratory syndrome coronavirus 2
PA: Physical activity
COMPASS: Cannabis use, Obesity, Mental health, Physical activity, Alcohol use, Smoking, and Sedentary behaviour
CESD-R-10: Center for Epidemiologic Studies Depression Scale (Revised)-10
GAD-7: Generalised Anxiety Disorder 7-item Scale
FLOURISH: Diener’s Flourishing Scale
ANOVA: Analysis of variance
